# CD8 positive T cells express IL-17 in patients with chronic obstructive pulmonary disease

**DOI:** 10.1186/1465-9921-12-43

**Published:** 2011-04-10

**Authors:** Ying Chang, Jessica Nadigel, Nicholas Boulais, Jean Bourbeau, François Maltais, David H Eidelman, Qutayba Hamid

**Affiliations:** 1 Meakins-Christie Laboratories and Respiratory Division, Department of Medicine McGill University, 3626 rue St. Urbain, Montreal, QC, H2X 2P2 Canada; 2 Respiratory Division, Research Institute of McGill University Health Centre, 2155 Guy Street, Suite 900 Montreal, QC, H3H 2R9 Canada; 3 Respiratory Division, Laval University, 2325 rue de l'Université, Québec, QC, G1V0A6 Canada

**Keywords:** Chronic Obstructive Pulmonary Disease, IL-17, Tc17 cells

## Abstract

**Background:**

Chronic obstructive pulmonary disease (COPD) is a progressive and irreversible chronic inflammatory disease of the lung. The nature of the immune reaction in COPD raises the possibility that IL-17 and related cytokines may contribute to this disorder. This study analyzed the expression of IL-17A and IL-17F as well as the phenotype of cells producing them in bronchial biopsies from COPD patients.

**Methods:**

Bronchoscopic biopsies of the airway were obtained from 16 COPD subjects (GOLD stage 1-4) and 15 control subjects. Paraffin sections were used for the investigation of IL-17A and IL-17F expression in the airways by immunohistochemistry, and frozen sections were used for the immunofluorescence double staining of IL-17A or IL-17F paired with CD4 or CD8. In order to confirm the expression of IL-17A and IL-17F at the mRNA level, a quantitative RT-PCR was performed on the total mRNA extracted from entire section or CD8 positive cells selected by laser capture microdissection.

**Results:**

IL-17F immunoreactivity was significantly higher in the bronchial biopsies of COPD patients compared to control subjects (*P *< 0.0001). In the submucosa, the absolute number of both IL-17A and IL-17F positive cells was higher in COPD patients (*P *< 0.0001). After adjusting for the total number of cells in the submucosa, we still found that more cells were positive for both IL-17A (*P *< 0.0001) and IL-17F (*P *< 0.0001) in COPD patients compared to controls. The mRNA expression of IL-17A and IL-17F in airways of COPD patients was confirmed by RT-PCR. The expression of IL-17A and IL-17F was co-localized with not only CD4 but also CD8, which was further confirmed by RT-PCR on laser capture microdissection selected CD8 positive cells.

**Conclusion:**

These findings support the notion that Th17 cytokines could play important roles in the pathogenesis of COPD, raising the possibility of using this mechanism as the basis for novel therapeutic approaches.

## Introduction

Chronic obstructive pulmonary disease (COPD), a progressive and irreversible chronic inflammatory disease of the lung caused predominantly by cigarette smoking, is one of the most important causes of mortality globally [1]. The inflammatory response in the lungs of COPD patients has been found to be strongly linked to tissue destruction and alveolar airspace enlargement, which lead to disease progression [2].

The inflammatory response reflects both the innate immune response to cigarette smoke exposure in the form of cellular infiltration by neutrophils and macrophages, as well as the adaptive immune response involving B and T cells, which is intimately linked with innate immunity [3]. COPD is marked by the accumulation of both CD4^+ ^and CD8^+ ^T cells in the alveolar walls, with CD8^+ ^cells predominating [4]. Recent findings concerning the innate and acquired immune responses in COPD have led to the suggestion that there is an autoimmune component to its pathogenesis. This notion is supported by the similarity of pathophysiological characteristics between COPD and several autoimmune diseases, including rheumatoid arthritis (RA), defects in phagocytosis and other modes of clearance of necrotic cells and subcellular particles, a deficiency of regulatory T cells and the presence of autoantibodies and autoreactive T cells [5].

The nature of the immune reaction in COPD raises the possibility that IL-17 and related cytokines may contribute to this disorder. Th17, a newly described subset of T cells, were suggested to play a role in RA and psoriasis. To date six IL-17 family members (IL-17A, IL-17B, IL-17C, IL-17D, IL-17E/IL-25 and IL-17F) and five receptors (IL-17RA, IL-17RB, IL-17RC, IL-17RD and IL-17RE) have been identified, which are conserved in rodents and humans [6]. IL-17A and IL-17F display high sequence homology and can be secreted as homodimers, as well as IL-17A/F heterodimers, by both mouse and human cells [7,8]. Although IL-17 has been closely associated with a subset of T helper cells known as Th17 cells, γδ T cells, natural killer [9] T cells and neutrophils have also been shown to produce IL-17A in the lung [10]. IL-17 secretion triggers production of numerous chemokines, resulting in neutrophil and macrophage recruitment and subsequent pathogen clearance, thus IL-17 mediates cross-talk between the adaptive and innate immune systems, allowing for orchestration of an effective immune response [10,11].

Numerous studies demonstrated the importance of IL-17 in the context of autoimmunity [10], however little is known about IL-17 production in COPD. A recent study showed that IL-17A could induce production of mucin (MUC)5AC in human bronchial epithelial cells [12], supporting the potential involvement of IL-17A in the phenotypic manifestations of COPD. In addition, transgenic over expression of Il-17 in the alveoli of murine lung induces lung inflammation with a COPD-like phenotype [13]. Aside from IL-17A, IL-17F mediated pathways might also provide a link between local activation of T cells and sustained accumulation of neutrophils in inflamed airways [14]. A case-control study demonstrates an association between an IL-17F gene polymorphism and chronic inflammatory lung diseases, including bronchial asthma and COPD, suggesting that IL-17F may be critically involved in the pathogenesis of chronic inflammatory lung diseases [15].

A well-known hallmark of COPD is that it is relatively unresponsive to treatment with steroids. Corticosteroids alone have little impact on the cellular inflammation or increased protease burden observed in COPD [16]. In addition, whereas exogenous steroids are able to suppress cytokine production in cells collected from non-diseased airways, the same cell types from patients with COPD are resistant to steroid treatment [17]. In this regard, it is of interest that IL-17 expression has been associated with diminished steroid responsiveness [15]. Moreover, there has been a recent suggestion that autoimmunity plays a role in the pathogenesis of COPD [5] and given the increased expression of IL-17 in certain autoimmune diseases [10], this further raises the possibility of its involvement in the pathogenesis of COPD.

In the present study, we analyzed the expression of IL-17A and IL-17F as well as the phenotype of cells producing them in the bronchial biopsies from COPD patients using immunohistochemistry, immunofluorescence staining, laser capture microdissection and quantitative reverse transcription-PCR. For the first time, we demonstrated the IL-17A and IL-17F expression in CD4^+ ^and especially in CD8^+ ^T cells in the airways of COPD patients. We also showed higher expression of these cytokines in COPD patients compared to control subjects. This study supports the notion that IL-17 is a pathogenetic element of COPD and suggests the possibility that a strategy of targeting IL-17 as a therapeutic target may be of value in this disease.

## Methods

### Subjects

Bronchoscopic biopsies from the subsegmental bronchi were obtained from 16 clinical diagnosis of COPD patients (GOLD stage 1-4) and 15 control subjects using published techniques [18] at the Montreal Chest Institute of the McGill University Health Centre and Laval Hospital, Canada. The COPD patients were eligible for this study if they met the following criteria: age ≥ 40 and ≤ 75 years; smoking history (≥ 10 pack-years); post-bronchodilator FEV_1_≥ 25% of predicted value and post-bronchodilator FEV_1_/forced vital capacity (FVC) ≤ 0.70; no history of asthma, atopy (as assessed by an allergy skin prick test during screening) or any other active lung disease. Patients on home oxygen or with raised carbon dioxide tension (>44 mmHg), α_1_-antitrypsin deficiency, recent exacerbation (in the last 4 weeks), uncontrolled medical condition or hypersensitivity to inhaled corticosteroids and bronchodilators were not eligible for the study. The experimental procedures were performed with ethical approval from the Research Ethics Boards of the McGill University Health centre and Laval University (Table 1).

**Table 1:** Clinical characteristics of COPD and control subjects

	COPD	Controls
Number	16	15
Age	53 ± 6	48 ± 9
Male/Female	10/6	11/4
Current/ex-smokers	7/8	0/3
Post-BD FEV1% predicted	60 ± 18	95 ± 12
TLCO%	60 ± 15	100 ± 20
GOLD Stage		
I	2	-
II	8	-
III-IV	6	-
Respiratory Medication		
SABD	13	-
LABD	4	-
ICS	5	-
Combination (LABD+ICS)	3	-
Theophylline	0	-

Data are presented as mean ± SD. BD, bronchodilator; FEV1, forced expiratory volume in 1s; TLCO, Transfer Factor of the Lung for Carbon Monoxide; SABD, short-acting bronchodilators; LABD, long-acting bronchodilators; ICS, inhaled corticosteroids.

### Processing of airway biopsies

Duplicate biopsy specimens from each case were immediately fixed in 4% paraformaldehyde for 4 h, and then treated in PBS/DEPC for overnight at 4°C. One specimen was dehydrated in alcohol and xylol and embedded in paraffin for immunohistochemistry, which was carried out on 5 μm thick sections. The second was snap-frozen in liquid nitrogen-cooled isopentane for immunofluorescence (6 μm thick), laser capture microdissection and quantitative reverse transcription-PCR studies (10 μm thick).

### Immunohistochemistry

Paraffin-embedded specimens were deparaffinized in xylene, rehydrated through a decreasing ethanol gradient, and rinsed in PBS. Antigen unmasking was performed with 10 Mm citrate buffer pH 6 and following with 0.2% Triton X100 in PBS. Endogenous peroxidase activity was blocked with 6% hydrogen peroxide for 30 min at room temperature. The slides were washed and pretreated with universal blocking solution (Dako, Carpinteria, USA). Slides were incubated overnight at 4°C using diluted goat anti-human IL-17A (AF317-NA, R&D Systems) or IL-17F (AF1335, R&D Systems) polyclonal antibodies or relevant isotype controls (AB-108-C, R&D Systems). The slides were rinsed and incubated with a biotinylated secondary antibody for 30 min at room temperature. After washing in PBS, the complex Streptavidin/HorseRadish Peroxidase (Vector) was applied for 30 min at room temperature. The reaction result was visualized with DAB/hydrogen peroxide (DAB Kit, Dako). The sections were finally rinsed in distilled water, lightly stained with hematoxylin, dehydrated, cleared, and cover slipped. Sample processed the same isotypes as primary antibody served as negative control.

### Immunofluorescence double staining

After permeabilization in PBS-Triton X100 0.2% for 10 min at room temperature, the sections were blocked with the universal blocking solution (Dako) for 30 min. The sections for double labeling with IL-17A or IL-17F paired with CD4 and CD8 respectively were incubated with diluted goat anti-human IL-17A antibody (1:100) or IL-17F antibody (1:200) (R&D Systems) paired with mouse anti-human CD4 antibody (1:40) (VP-C319, Vector), CD8 antibody (1:120) (M7103, Dako) or relevant isotype controls (MAB002, R&D Systems) for overnight at 4°C. After rinsing with PBS, the sections were then reacted with Alexa 488-conjugated rabbit anti-goat IgG and Alexa 555-conjugated rabbit anti-mouse IgG (Molecular probes Inc., Eugene, OR), diluted together at 1:300 in PBS for 30 min. The sections were then cover slipped with PermaFluor Aqueous mounting medium (Thermo; Pittsburgh, PA). Fluorescence immunolabeling signals were detected by a fluorescence microscope (Olympus BX51TF, Japan).

### Laser Capture Microdissection

Laser capture microdissection (LCM) was performed using the PixCell II apparatus (Arcturus Biosciences, Moutain View, CA) in accordance with the manufacturer's instructions. A fast immunohistochemistry staining was performed on frozen tissue sections (10 μm). Briefly, after treated with blocking solution (Dako) and 0.5% Triton-X100, the sections were incubated with mouse anti-human CD8 (Ced) for 10 min and following with biotinylated rabbit anti-mouse IgG (Dako) for 10 min. Then the sections were incubated with streptavidin-HRP for 8 min and visualized with DAB/hydrogen peroxide. After counterstaining with haematoxyline, sections were dehydrated in increasing ethanol gradient and 100% xylene immediately before performing LCM. The labeled cells were captured by LCM. For each sample, LCM was performed on 8 to 10 tissue sections yielding approximately 300 to 500 cells per section. The sections were pooled to yield approximately 3000 to 5000 cells per sample. As CD4^+ ^cells were already known to express IL-17 [17,19], this part of study was done to confirm the expression of IL-17 in CD8^+ ^cells.

### RNA Isolation and Quantitative Reverse Transcription-PCR

Total RNA was isolated using the RNeasy Micro RNA isolation kit (Qiagen) from LCM samples or RLT lysis buffer (Qiagen) with 1% β-mercaptoethanol treated airway tissues from entire frozen sections. Complementary DNA was synthesized by reverse transcription (RT) of total isolated RNA (Superscript II First Strand Synthesis, Invitrogen, Carlsbad, CA). Quantitative RT-PCR for IL-17A, IL-17F and glyceraldehyde-3-phosphate dehydrogenase (GAPDH) as performed using a Step One Plus Thermal Cycler (Applied Biosystems, Foster City, CA) with Power SYBR Green PCR Master Mix (Applied Biosystems). The primers used for the specific amplified genes of IL-17A (174 bp), IL-17F (200 bp) and GAPDH (139 bp) are as follows:

IL-17A forward: 5'-CATCCATAACCGGAATACCAATA-3'; IL-17A reverse: 5'-TAGTCCACGTTCCCATCAGC-3'; IL-17F forward: 5'-GTGCCAGGAGGTAGTATGAAGC-3'; IL-17F reverse: 5'-ATGTCTTCCTTTCCTTGAGCATT-3'; GAPDH forward: 5'-AGTCAACGGATTTGGTCGTATT-3'; GAPDH reverse: 5'-ATGGGTGGAATCATATTGGAAC-3';

### Analysis for immunohistochemistry

Immunostained cells in the airway submucosa were counted at a magnification of 400. The area of submucosa was measured by using the software Image Pro 6.2 (MediaCybernetics, Bethesda, USA). The final result was expressed as the number of positive cells/mm^2^. The number of cells was corrected for the total number of cells by counting the number of nuclei in the submucosa. The positive staining area of IL-17F in airway epithelium was measured and the results were presented as the percentage of positive area in total epithelium area.

### Statistical analysis

Data were expressed as median (range). The mean value of IL-17A (in positive cells/mm^2^) and IL-17F (in positive cells/mm^2 ^or positive area percentage) in the COPD patients and in the normal controls were analyzed using Mann-Whitney U test. Probability values of *P *< 0.05 were considered significant. Data analysis was performed by using the Graphpad Instat 3 software (GraphPad Software, La Jolla, California).

## Results

### IL-17A and IL-17F expression is increased in airways of COPD patients

**Figure 1:**
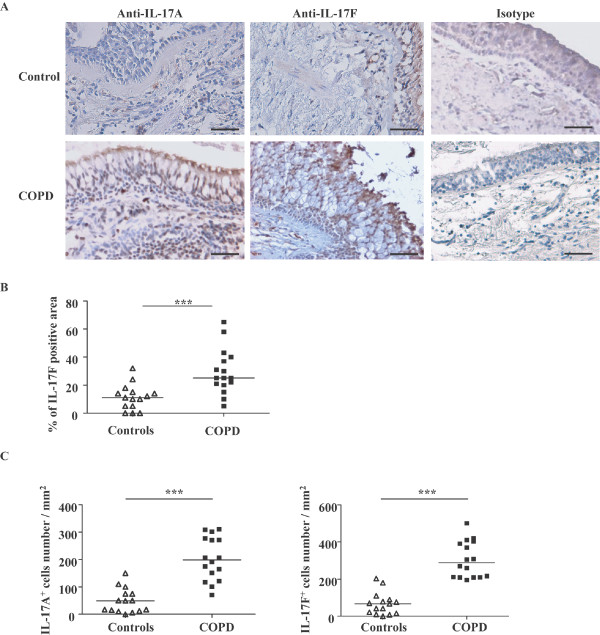
**IL-17A and IL-17F expression in COPD patients**. **(A) **Immunohistochemistry, positive staining appears brown color. Magnification, 100 ×. Scale bar = 50 μm. **(B) **IL-17F expression in epithelium of airways of COPD patients. IL-17F positive area in epithelium was measured as outlined in text. **(C) **IL-17A and IL-17F expression in submucosa of airways of COPD patients. Absolute IL-17A^+ ^and IL-17F^+ ^positive cells in submucosa were counted. Results are expressed as median (range), n = 15 and 16 subjects for controls and COPD patients respectively. ***P < 0.0001.

We observed IL-17A and IL-17F expression in the airways of control subjects and COPD patients by immunohistochemistry. We were only able to detect occasional immunoreactivity of IL-17A expression in epithelium. In contrast, we observed considerable staining for IL-17F in airway epithelium (Figure 1A), which was greater in the airways of COPD subjects compared to controls (25 (5-65) % vs. 11 (0-32) %, *P *< 0.0001) (Figure 1B). In the submucosa, both IL-17A and IL-17F positive cells were observed (Figure 1A), and the absolute number of cells expressing both of these cytokines was higher in COPD subjects than in control subjects (IL-17A^+^: 199 (70-310) vs. 49 (0-150); IL-17F^+^: 287 (195-501) vs. 67 (0-203); *P *< 0.0001) (Figure 1C). There was also some immunoreactivity of IL-17A and F in the endothelial site of few blood vessels in some sections.

### More submucosal cells expressed IL-17A and IL-17F in airways of COPD patients

**Figure 2:**
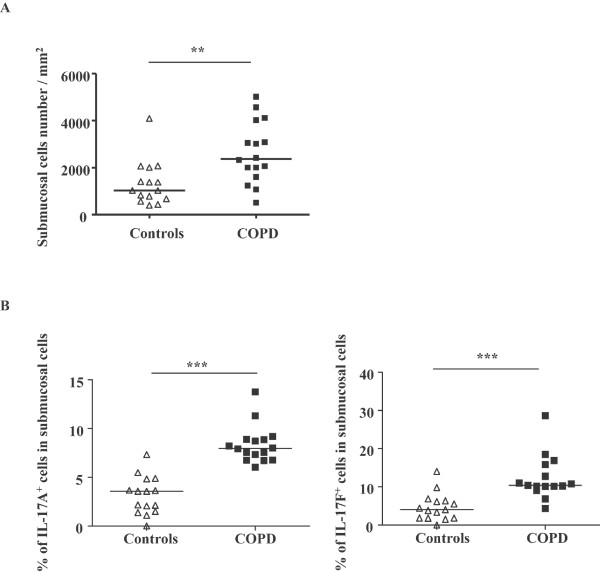
**Percentage of IL-17A^+ ^and IL-17F^+ ^cells in airway submucosal cells of COPD patients**. **(A) **Submucosal cells in airways of COPD patients. **(B) **Percentage of IL-17A^+ ^and IL-17F^+ ^cells in airway submucosal cells of COPD patients. Results are expressed as median (range), n = 15 and 16 subjects for controls and COPD patients respectively. **P < 0.001, ***P < 0.0001.

As expected, we found that the number of submucosal cells in the airways of COPD subjects was greater than in control subjects (2371 (509-5011) vs. 1025 (391-4087), *P *< 0.001) (Figure 2A). We therefore evaluated the relative number of IL-17A^+ ^and IL-17F^+ ^cells taking in consideration the total number of submucosal cells. Similarly there was greater number of cells positive for both IL-17A and IL-17F in COPD subjects compared to controls (IL-17A: 8 (6-14) % vs. 3 (0-7) %, *P *< 0.0001; IL-17F: 10 (4-28) % vs. 4 (0-14) %, *P *< 0.0001) (Figure 2B).

### IL-17A and IL-17F expression is not regulated on transcriptional level

**Figure 3:**
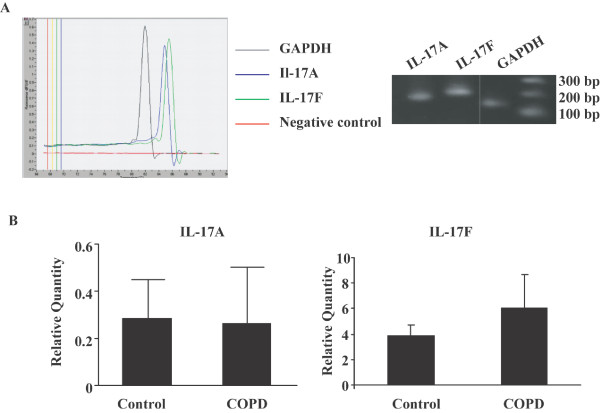
**IL-17A and IL-17F mRNA expression in airways of COPD patients**. **(A) **Quantitative RT-PCR was performed from frozen airways sections of COPD patients. One representative example from 7 subjects with similar results is shown. **(B) **Quantification of IL-17A and IL-17F mRNA expression in airways of control subjects and COPD patients. Results are expressed as means ± SEM. N = 7 for both control subjects and COPD patients.

To further investigate the expression of IL-17A and IL-17F in COPD, we performed quantitative RT-PCR on frozen airways sections of COPD patients. As with protein expression, the expression of IL-17A and IL-17F mRNA was also detected in airways of COPD patients (Figure 3A). Although there was trend for IL-17F to be more increased in COPD patients compared to control, the quantification of IL-17A and IL-17F mRNA in COPD patients was not statistically higher compared to control (Figure 3B).

### IL-17A and IL-17F expressed in CD4^+ ^and CD8^+ ^T cells

**Figure 4:**
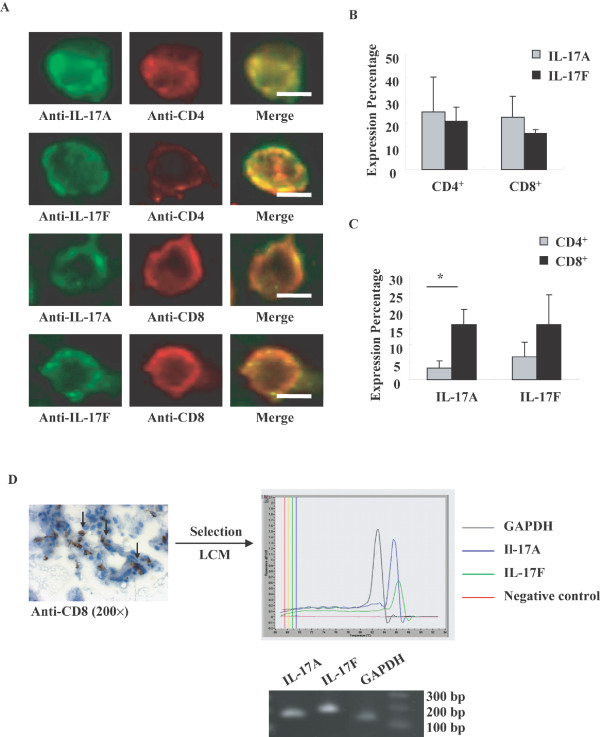
**Double immunofluorescence staining for detection of IL-17A and IL-17F expression in CD4^+ ^and CD8^+ ^T cells in airways of COPD patients**. **(A) **Double immunofluorescence staining was performed. Scale bar = 5 μm. **(B) **Percentage of CD4^+ ^and CD8^+ ^T cells that express IL-17A and IL-17F. Results are expressed as means ± SEM. **(C) **Percentage of CD4^+ ^and CD8^+ ^T cells that express IL-17A and IL-17F in total IL-17A^+ ^and IL-17F^+ ^cells. Results are expressed as means ± SEM. *P < 0.05. N = 3 COPD patients. **(D) **IL-17A and IL-17F mRNA expression in CD8^+ ^T cells in airways of COPD patients. Immunohistochemistry determined CD8^+ ^T cells were selected by LCM, and then RT-PCR was performed to detect the mRNA expression of IL-17A and IL-17F. One representative result from 3 subjects is shown.

To investigate the relationship of IL-17A&F to T cells, we used double immunofluorescence staining with antibodies to IL-17A or IL-17F and antibodies to CD4^+ ^or CD8^+ ^T cells. In the airways of COPD subjects, both CD4^+ ^and CD8^+ ^T cells expressed IL-17A and IL-17F (Figure 4A). To our knowledge, this is the first demonstration that CD8^+ ^cells produce IL-17A and IL-17F in COPD. Furthermore, we estimated the percentage of CD4^+ ^and CD8^+ ^T cells that express IL-17A and IL-17F as well as the percentage of IL-17A^+ ^and IL-17F^+ ^cells that co-express T cell markers. In COPD patients, similar percentage of CD4^+ ^and CD8^+ ^T cells that express IL-17A and IL-17F was observed (Figure 4B). While in total IL-17A^+ ^cells, the percentage of IL-17A positive cells that co-express CD8 immunoreactivity was significantly higher than that expressing CD4^+ ^T cells (16.0 ± 4.3% vs. 3.4 ± 2.0%, P < 0.05, Figure 4C). A similar trend was also observed in total IL-17F^+ ^cells (15.8 ± 8.8% vs. 6.6 ± 4.3%, Figure 4C). We further confirmed this finding using LCM to select CD8^+ ^T cells for the detection of expression of IL-17 mRNA by RT-PCR (Figure 4D).

## Discussion

This study aimed to investigate the possibility that the Th17 cytokines including IL-17A and IL-17F are involved in the pathogenesis of COPD. Using bronchial biopsies from COPD patients, we found evidence that the expression of both IL-17A and IL-17F is increased in the airways of COPD subjects in both inflammatory cells as well as the airway epithelium. These observations add to the growing evidence, which suggests that Th17 cytokines play a significant role in this disease.

Using immunocytochemistry, we consistently detected increased expression of both IL-17A and IL-17F in the airways of COPD patients compared to controls. Both cytokines were present to a much greater extent than in controls (Figure 1). The pattern of expression appeared to differ between the two cytokines in that we detected IL-17A and F in the epithelium of COPD patients but very little in controls. However, the best control group is smokers without COPD, but we were unable to obtain such a group.

The detection of considerable level of IL-17F in the epithelium is of interest given the potential importance of the epithelium in the inflammatory process of COPD [20]. When stimulated with pro-inflammatory mediators, the airway epithelium releases chemoattractants CXCL1 (GRO-α), CXCL5 (ENA-78), CXCL6 (GCP-2), CXCL8 (IL-8) and CCL5 (RANTES)[21,22]. Overexpression of IL-17F predominantly expressed in bronchial epithelial cells has also been reported in ovalbumin challenged mice [23]. In addition, overexpression of IL-17F in murine lung epithelium leads to infiltration of lymphocytes and macrophages and mucus hyperplasia [24]. Taken together, these observations suggest the possibility that IL-17F contributes to amplification of the ongoing inflammatory processes not only through the recruitment and activation of specific subset of inflammatory cells, but by prolonging their survival in the airway.

Our results contrast to some degree with the recent report of Di Stefano et al [25] who found evidence of increased production of IL-17A but not IL-17F in the bronchial submucosa of COPD patients. Furthermore, they detected expression of both IL-17A and IL-17F in the epithelium but failed to detect a difference between controls and COPD patients. The discrepancy between their results and ours may reflect differences in patient selection or technique. Notwithstanding these differences, reports to date consistently support the notion that there is increased expression of IL-17A and IL-17F in COPD patients, underscoring the potential importance of Th17 cytokines in this disease.

A potential explanation of increased expression of IL-17 in COPD airways is that this may be simply a reflection of the presence of greater numbers of submucosal cells. Indeed, consistent with previous studies, we detected increased cell number in the airway submucosa of COPD patients (Figure 2). However, even after accounting for this, we still detected significant differences between COPD and control, as the proportion of submucosal cells expressing IL-17A and IL-17F in COPD subjects was greater than that in controls. To further explore the basis for this increased expression by submucosal cells, we undertook studies of cytokine expression at the mRNA level. As expected, we were able to consistently detect evidence of IL-17A and IL-17F mRNA in the airways of COPD subjects (Figure 3). However the quantification results showed that the mRNA expression of IL-17A and F was not statistically different between COPD patients and controls, suggesting that there is a discrepancy between mRNA and protein expression for IL-17A and F in COPD patients and that increased IL-17A expression in COPD patients is regulated at translational level. To refine this observation, we employed a combination of immunocytochemistry and laser capture microscopy. Double immunostaining demonstrated detection of IL-17A and IL-17F not only in CD4^+ ^cells as expected, but also in CD8^+ ^cells (Figure 4). The high percentage of IL-17A and IL-17F expressing CD immunoreactivity suggested that CD8^+ ^T cells are major source of these cytokines particularly in COPD [4]. We then used laser capture microscopy to select regions of the airway that were positive for either CD4 or CD8 by immunostaining from which we extracted the RNA to confirm that both CD4^+ ^and CD8^+ ^cells express IL-17A and IL-17F mRNA (Figure 4). To our knowledge, this is the first definitive demonstration that both CD4^+ ^and CD8^+ ^cells are capable of expressing Th17 cytokines in COPD. COPD is marked by increased number of T cells in lung parenchyma and both peripheral and central airways, with a greater increase in CD8^+ ^cells relative to CD4^+ ^T cells [4]. A number of studies have attempted to characterize the pattern of lymphocyte cytokine production in COPD, but the results are conflicting [18,26]. Nevertheless, in the context of this observation it is noteworthy that a recent study has reported that CD8^+ ^T cells are activated in the presence of the cytokines IL-6 or IL-21 plus TGF-β, develop into IL-17-producing (Tc17) cells. Our findings also need to be taken seen in the context of reports of Tc17 cells in a variety of immunological diseases. For example, Tc17 have also been found in cutaneous inflammatory diseases like psoriasis vulgaris [27] and allergic contact dermatitis [28]. Tc17 cells may also be important in defense against viruses [29,30].

The observation that expression of Th17 cytokines is increased in COPD raises questions as to how this may come about. The combination of IL-6 and TGF-β is reported to skew the balance of T helper cells toward Th17 cell differentiation [31]. In this regard, it is of interest that increased production of IL-6 and TGF-β has been reported in COPD patients [32], raising the possibility that IL-6 and TGF-β may enable the promotion of Th17 cells differentiation in COPD. Regardless of the mechanism, Th17 cytokines have the potential to contribute to COPD in various ways. IL-17A acts directly on epithelial cells and on airway fibroblasts and smooth muscle cells to induce the secretion of neutrophil-recruiting chemokines, such as CXCL8 [31]. Although a comprehensive comparative analysis of IL-17F and IL-17A has not been performed, IL-17F appears to have biological actions similar to IL-17A both *in vitro *and *in vivo *[14]. Therefore it is possible that with activation of IL-17A and IL-17F mediated pathways, a crosstalk between local activation of T cells and sustained accumulation of neutrophils in inflamed airways could be established. Zhu et al [33] have suggested that biopsies from patients with chronic bronchitis have more inflammation compared to patients with COPD but without chorionic bronchitis. This group of patients might have more IL-17 expression. However in our study we did not group our subjects and presented the data of our patients as one group according to GOLD classification.

In summary, in bronchial biopsies we detected clear evidence that the expression of the cytokines IL-17A and IL-17F is increased in COPD compared to control. In the case of IL-17F, this increased expression extends to the epithelium and is not simply restricted to the submucosa. Most importantly, we detected increased expression of these cytokines in both CD4^+ ^and CD8^+ ^cells, suggesting that the inflammatory process in COPD may resemble that in other disorders where Tc17 cells are active. These findings contribute to the growing body of information that supports the importance of investigating the role of IL-17 and related cytokines in COPD, potentially providing novel therapeutic targets in this important chronic disease.

## Competing interests

The authors declare that they have no competing interests.

## Authors' contributions

YC carried out the cell counting and data analysis and drafted the manuscript. JN performed the RT-PCR. NB carried out the immunohistochemistry staining and laser capture. JB and FM participated in the sample collection and did the immunocytochemiostry. DHE participated in the design of the study and corrected the manuscript. QH supervised of the study. All authors read and approved the final manuscript.
